# Thermal Characterization of Low-Dimensional Materials by Resistance Thermometers

**DOI:** 10.3390/ma12111740

**Published:** 2019-05-29

**Authors:** Yifeng Fu, Guofeng Cui, Kjell Jeppson

**Affiliations:** 1 Electronics Materials and Systems Laboratory, Department of Microtechnology and Nanoscience, Chalmers University of Technology, SE-41296 Gothenburg, Sweden; kjell.jeppson@chalmers.se; 2 Key Laboratory for Polymeric Composite & Functional Materials of Ministry of Education, The Key Lab of Low-Carbon Chemistry and Energy Conservation of Guangdong Province, Materials Science Institute, School of Chemistry, Sun Yat-sen University, Guangzhou 510275, China; cuigf@mail.sysu.edu.cn

**Keywords:** thermal characterization, resistance temperature detector, heat spreader, carbon nanotube, graphene, boron nitride

## Abstract

The design, fabrication, and use of a hotspot-producing and temperature-sensing resistance thermometer for evaluating the thermal properties of low-dimensional materials are described in this paper. The materials that are characterized include one-dimensional (1D) carbon nanotubes, and two-dimensional (2D) graphene and boron nitride films. The excellent thermal performance of these materials shows great potential for cooling electronic devices and systems such as in three-dimensional (3D) integrated chip-stacks, power amplifiers, and light-emitting diodes. The thermometers are designed to be serpentine-shaped platinum resistors serving both as hotspots and temperature sensors. By using these thermometers, the thermal performance of the abovementioned emerging low-dimensional materials was evaluated with high accuracy.

## 1. Introduction

The semiconductor industry is pursuing electronic systems with higher integration density, more functions, higher power and frequency, and smaller footprint and volume, with lower cost. When the performance increases, the power density in electronics systems becomes higher and higher; thus, heat dissipation becomes a critical issue. In addition, the increase of hotspots and packaging complexity, such as in three-dimensional (3D) stacking of processor and memory chips, makes thermal management an even more difficult task in microsystems. Various advanced materials and technologies were proposed and demonstrated to improve thermal management in electronics, for instance, nanoparticles and graphene-enhanced thermal interface materials (TIMs) [1], carbon nanotube (CNT)-based TIMs [2], cooling fins [3], etc. Therefore, thermal characterization of such nanomaterials and nanostructures becomes more important than ever to evaluate their performance.

Various methods were developed to characterize the thermal performance of nanomaterials. For instance, the thermal bridge method can be used to measure the in-plane thermal conductivity of extremely small structures down to a single atom layer [4]; the e-beam self-heating method can be used to measure the contact thermal resistance at material interfaces [5]; scanning thermal microscopy is able to map local temperature with nanoscale resolution and thermal conduction in materials [6]; the optothermal Raman spectroscopy technique allows high accuracy measurement of the thermal conductivity of atomic thick nanomaterials [7]; the pulsed photothermal reflectance method can be used to measure both thermal conductivity of materials and contact thermal resistance at interfaces [8]; the 3ɷ method allows high accuracy measurement of the thermal conductivity of materials [9]; the transient plane source method allows fast measurement of thermal conductivity, thermal diffusivity, and specific heat capacity of materials [10]; the laser flash method is also an easy-to-implement method for thermal conductivity measurement of materials [11]. It should be noted that the method should be selected depending on the size, geometry, composition, and performance of the materials in order to perform a proper characterization. Among all the thermal characterization methods, the on-chip resistance thermometer is a component allowing high accuracy, high speed, and real-time characterization of nanomaterials and nanostructures. This paper is expanded from a conference paper [12] but elaborates upon and includes the most recent published results to review the previous work on thermal characterization of various one- and two-dimensional (1D and 2D) nanomaterial-based cooling structures using resistance thermometers. First of all, the design, fabrication, and calibration of the resistance thermometer is presented. Secondly, we summarize the thermal characterizations of different low-dimensional materials using the resistance thermometer. This includes CNT-based cooling fins, graphene-based lateral heat spreaders, and boron nitride (BN)-based heat spreaders.

## 2. Resistance Thermometers

**Figure 1:**
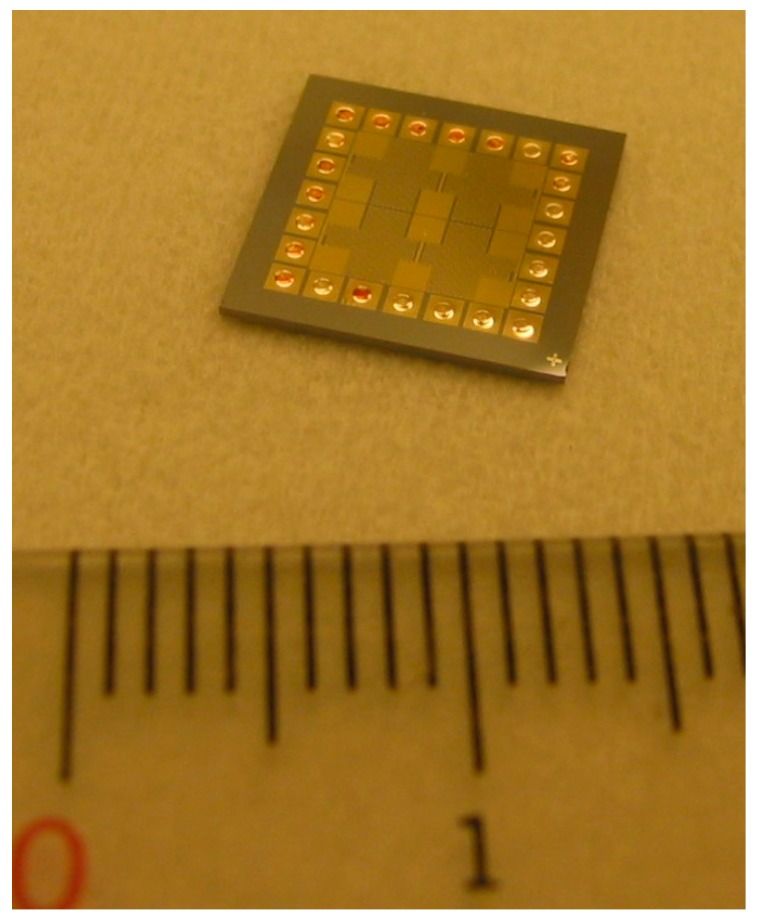
The thermal test chip with resistance thermometers and heating elements.

The principle of a resistance thermometer is to use temperature-sensitive materials to detect temperature by monitoring the change in electrical resistance of the material. Among all the materials, platinum (Pt) is one of the most used due to its highly linear temperature–resistance relationship. Fu et al. fabricated a resistance thermometer using e-beam evaporated Pt thin films on silicon chips [13], as shown in Figure 1. In order to realize the temperature monitoring in an embedded interface, they used through-silicon via (TSV) technology to read out the temperature. The serpentine Pt temperature sensors can also simultaneously act as heating elements to simulate hotspots in chips for the thermal characterization of heat dissipation materials and structures. After the fabrication, the resistance thermometers were calibrated by a standard resistance temperature detector (RTD). After calibration, the resistance thermometers can be used to monitor the temperature distribution on the thermal test chip; therefore, the cooling performance can be easily evaluated by simply measuring the resistance.

The thermal test chip fabricated by Fu et al. [13] shown in Figure 1 consists of a 3 × 3 array of thermometers with a size of 390 × 400 µm^2^. The thickness of the platinum thermometers is 40 nm. Prior to the deposition of the platinum resistors, a 20-nm-thick titanium layer was deposited as an adhesion layer. The thickness of the insulating silicon dioxide (SiO_2_) layer on the silicon substrate was 300 nm. Balandin et al. used a similar structure to model the heat spreading from metal–oxide–semiconductor (MOS) field-effect transistors on silicon-on-insulator (SOI) substrates with and without graphene heat spreaders [14]. 

In this paper, the thermal test chip shown in Figure 1 and its slightly modified version (to provide even higher power density) were used to evaluate the cooling performance of various nanomaterials and nanostructures, and the results are presented in Section 3, Section 4 and Section 5. 

## 3. CNT-Based Micro Heat Sinks

**Figure 2:**
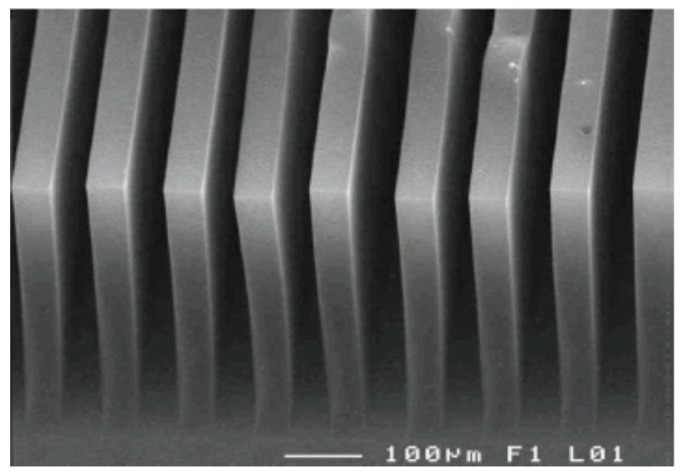
As-grown CNT cooling fins used to cool down the power transistor. Reprinted with permission from [3].

**Figure 3:**
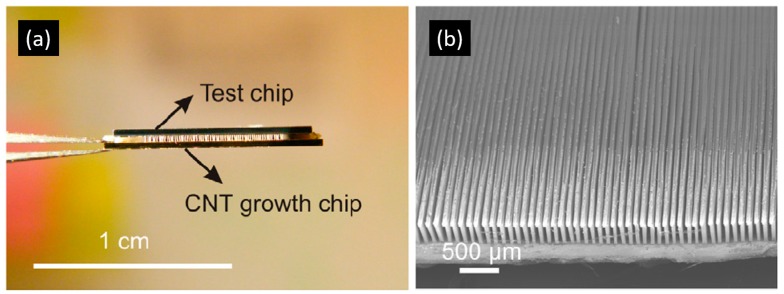
Transferred CNT cooling fins directly fabricated on top of the hotspot test structure. Reprinted with permission from Reference [22].

Owing to the very strong *sp^2^* hybridized C–C bonding, CNTs exhibit excellent thermal properties. Therefore, they were proposed as a candidate for thermal interface material development and many results were reported [15,16,17,18,19]. On the other hand, since CNTs are mechanically strong [20,21] and can be vertically aligned, they can also be applied as heat sinks. CNT-based micro heat sinks were demonstrated to cool down power transistors by Mo et al. [3]. They grew CNTs on a silicon chip (as shown in Figure 2) and fabricated the cooler separately before attaching it onto the power transistor. It was found that the CNT-based cooler was able to cool down the power transistor to a much lower temperature (108 °C vs. 119 °C) even at much higher power input (25.7 W vs. 19.6 W). Fu et al. modified the design and fabricated the CNT cooling fins directly on top of the hotspots on silicon chips in order to further decrease the thermal resistance on the heat dissipation path [22], as shown in Figure 3. They firstly grew the CNT structures on a silicon substrate using Fe as a catalyst, and then transferred the CNT cooling fins onto a thermal test chip with high-power-density hotspots. Low-melting-point metal indium was used as the transfer media so that the transfer process would be compatible with complementary metal–oxide–semiconductor (CMOS) processes. The CNT fin structure was electrically insulated from the hotspot resistor by a 300-nm SiO_2_ insulating layer on the hotspot circuit. More details about the transfer process can be found elsewhere [23,24]. Prior to the fabrication of the CNT cooling fins, multi-scale modeling was performed to optimize the dimension of the CNT structures (i.e., height, width, and pitch of the CNT fins); therefore, optimal pressure decrease (between coolant inlet and outlet) and maximal cooling effect were obtained.

**Figure 4:**
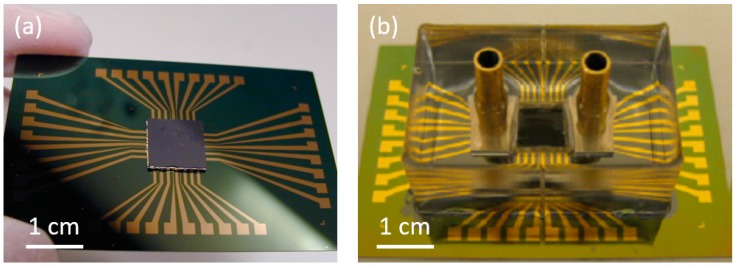
(**a**) On-chip CNT cooling fin test structure mounted on a supporting circuit board. (**b**) Complete cooling system embedded in polydimethylsiloxane (PDMS) with inlet and outlet nozzles for the coolant.

After transfer, the on-chip CNT-based micro heat sink was mounted onto a supporting circuit board as shown in Figure 4. To complete the cooling system, inlet and outlet nozzles were fabricated and connected to the CNT cooling fin structures through aluminum chambers at two ends of the test chip. Finally, polydimethylsiloxane (PDMS) was used to encapsulate the whole system to prevent coolant leakage. As a reference for studying the cooling performance of the CNT-based micro heat sink, identical cooling systems without CNT cooling fins were also fabricated and characterized.

**Figure 5:**
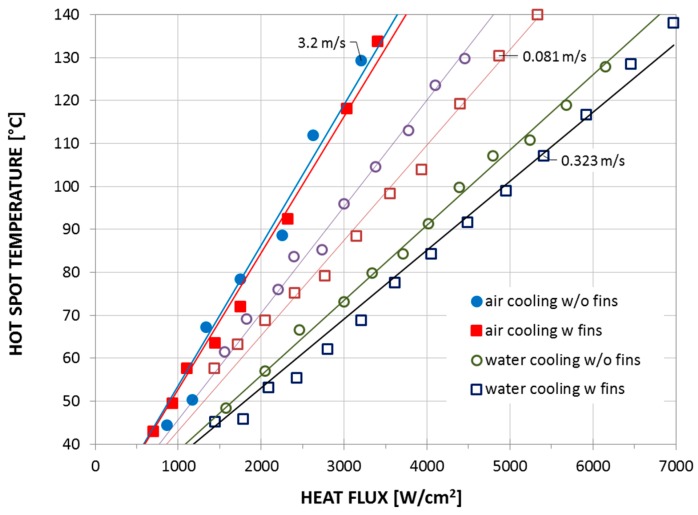
Cooling performance of the CNT-based micro heat sink using water as coolant (with air cooling as a reference) plotted as the hotspot temperature vs. heat flux. Experimental data sourced from Reference [22].

In order to examine the cooling performance of the CNT-based micro heat sink, air and water were used as coolant, and they were pumped to flow through the micro channels between the CNT fins. Some results of the experiments are shown in Figure 5 where the temperature at the hotspot is plotted vs. heat flux through the resistive hotspot. 

As expected, water is a much more effective coolant than air. For a heat flux of 3000 W/cm^2^, the hotspot temperature decreased by almost 50 °C (from 116 to 68 °C) upon using water at a flow rate of 0.32 m/s, compared to when air was used as the coolant, even though the air flow rate was ten times larger (3.2 m/s). However, more interesting is the unfortunate fact that the CNT cooling fins seemed to have a minimal influence when air was used as coolant. This is believed to be a combination of the thermal contact resistance to the hotspot being too high due to the interface layers, and that macro-scale cooling may not be directly scalable to a micro-scale environment.

**Figure 6:**
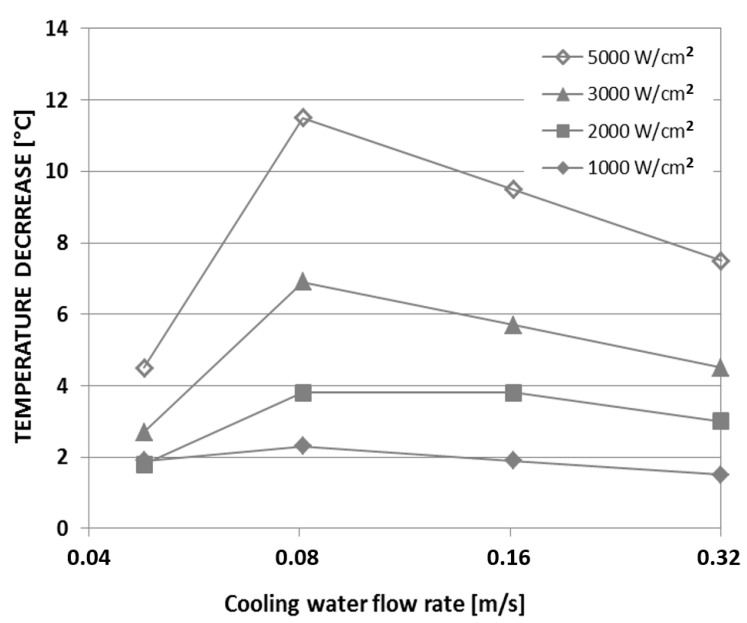
Hotspot temperature decrease vs. flow rate of water coolant for four different heat fluxes through the hotspot resistor. Experimental data sourced from Reference [22].

The experiments showed that, when the chip was cooled by water at a flow rate of 0.32 m/s, the hotspot temperature on the chip with the CNT cooling fin structure was about 8–10 °C lower than on the test chip without the CNT fins. Interestingly enough, beyond a certain flow rate of the water coolant, the cooling effect seems to be more or less independent of the flow rate, as shown in Figure 6. Since the water cooling of the indium adhesive seems to be so effective, the influence of the CNT cooling fins even appears to decrease as the flow rate of the water coolant increases beyond 0.08 m/s. 

**Figure 7:**
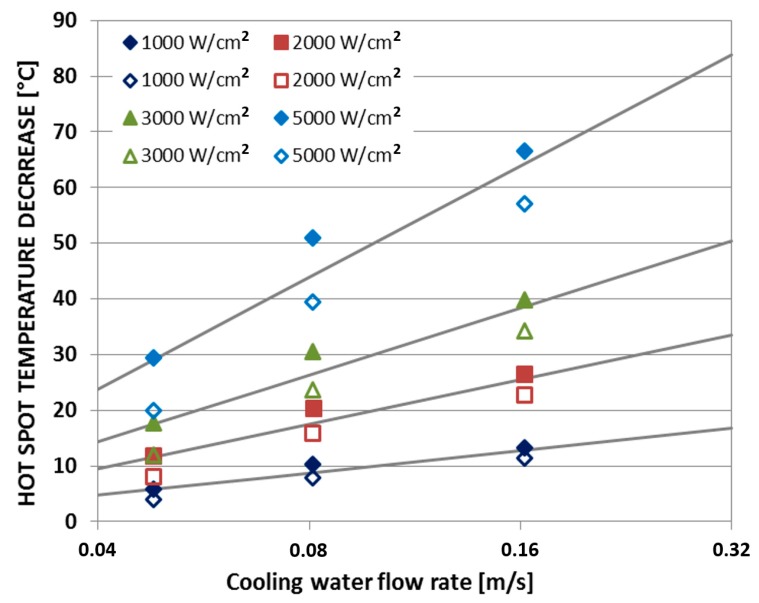
Hotspot temperature decrease vs. water coolant flow rate (test structure with CNT cooling fins = filled markers; test structure without CNT cooling fins = open markers). Experimental data sourced from Reference [22].

Finally, Figure 7 shows that the decrease of the hotspot temperature due to water cooling of the CNT fins seems to increase linearly with the flow rate of the water coolant. 

## 4. Graphene-Based Heat Spreaders 

Similar to CNTs, graphene also possesses excellent thermal and mechanical properties due to its special crystalline structure [25]. In electronic systems, non-uniform distribution of thermal energy dissipates from high-power components, such as high-power transistors and light-emitting diodes (LEDs), leading to the formation of hotspots, together with high average device temperatures, resulting in the degradation of device performance and poor reliability. Therefore, various thermal composites [26,27,28,29,30,31] and heat spreaders [8,32,33,34,35,36,37,38] were developed and demonstrated using liquid-phase exfoliated (LPE) graphene and chemical vapor deposition (CVD)-grown graphene.

**Figure 8:**
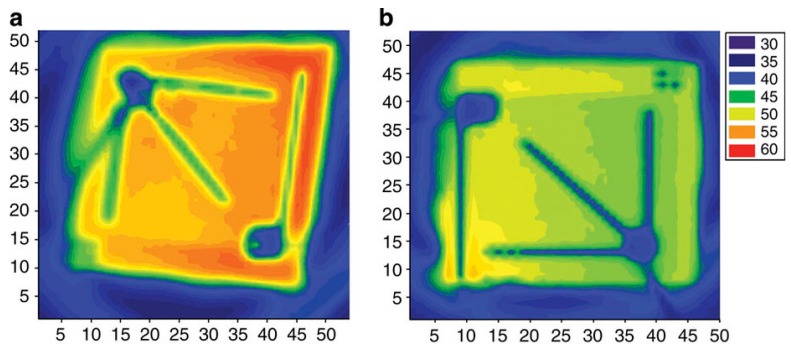
Infrared thermal imaging camera photographs of the chip surfaces showing the temperature distribution on the surface of (**a**) a conventional light-emitting diode (LED) chip, and (**b**) a reduced graphene oxide (rGO)-embedded chip under 100 mA current injections. Sourced from Reference [33]. Reprinted with permission from Nature Communications.

Balandin et al. showed that a few-layer graphene-based heat spreader connected to the drain of gallium nitride (GaN) high-power field-effect transistors considerably reduced the device temperature [32]. Using micro-Raman spectroscopy for in situ monitoring, they demonstrated that hotspot temperatures could be lowered by ∼20 °C in transistors operating at a power density of ~13 W per mm of channel width, which they claim corresponds to an order-of-magnitude increase in device lifetime. Similarly, Hong et al. showed improved heat dissipation in gallium nitride LEDs by embedding reduced graphene oxide (rGO) patterns into the devices [33]. The infrared images of the LED chip surfaces from their paper shown in Figure 8 indicate a decrease in peak temperature on the chip surface from 58 °C for a conventional LED to 53 °C for the rGO-embedded LEDs. In addition, the average temperature on the chip surface decreased from 51 to 47 °C. 

**Figure 9:**
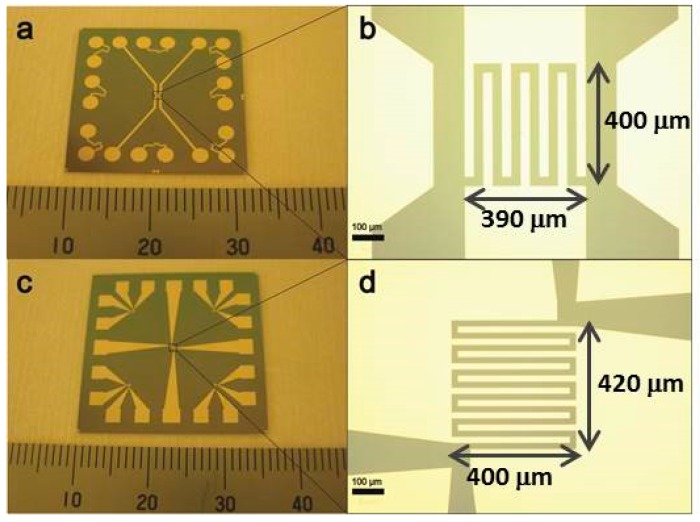
Redesigned resistance thermometers with larger area available for the heat spreader (**a**,**c**), and wider terminal wires (**b**,**d**).

To evaluate the graphene-based heat spreaders, a new version of the resistance thermometer was designed and fabricated. Based on the lessons learnt from the CNT-based micro heat sink, the wires connecting the hotspot resistor and the I/O pads were redesigned to minimize the power dissipation via interconnect circuit. Two examples of such redesigned resistance thermometers are shown in Figure 9.

**Figure 10:**
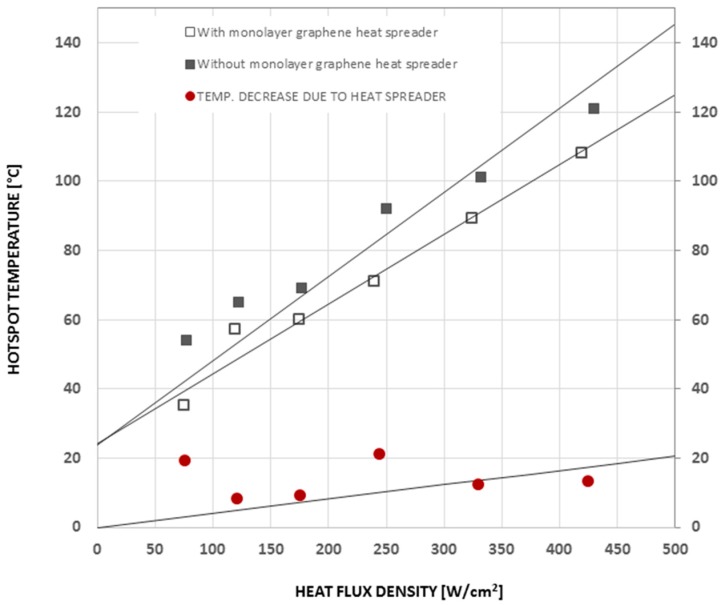
Temperature monitoring on thermal test chip with and without a monolayer graphene heat spreader. Replotted data sourced from Reference [34].

**Figure 11:**
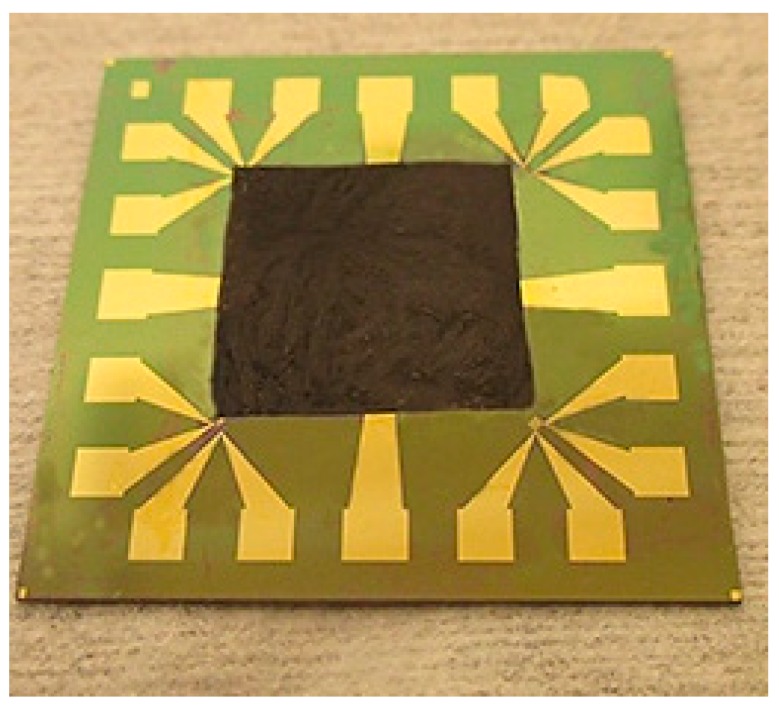
Multilayer transferred graphene film placed on hotspot test structure as a heat spreader across the chip surface.

These hotspot test structures were used in a series of experiments to investigate the thermal performance of 2D materials with high thermal conductivity, such as monolayer and multilayer graphene, and BN-based heat spreaders. By using such 2D materials as heat spreaders to dissipate the Joule heat generated from the hotspot laterally across the chip surface, both the hotspot temperature and the average temperature across the chip can be lowered. The area of the hotspot resistor used in these experiments was 390 × 400 μm^2^, and its resistance was about 80 Ω at room temperature. monolayer graphene grown by chemical vapor deposition (CVD) was placed on the thermal test chip as heat spreader via the transfer method [34,35]. The graphene was isolated from the resistor by a 30 nm SiO_2_ protective layer. Figure 10 shows the temperature vs. heat flux at the hotspot. It can be seen that the hotspot temperature can be decreased by about 10 °C by the graphene-based heat spreader (from 133 °C to 123 °C) at a heat flux of 460 W/cm^2^. Thick graphene-based films fabricated from the liquid-phase exfoliation method [8,36,37] were also applied as heat spreaders in the same way as the monolayer graphene as shown in Figure 11. To decrease the thermal contact resistance, the thickness of the SiO_2_ layer was reduced to one-tenth of the thickness that was used in the CNT cooling fin experiments. The detailed process of transferring and placing the monolayer and multilayer graphene heat spreader onto the hotspot structure is described elsewhere [34]. 

**Figure 12:**
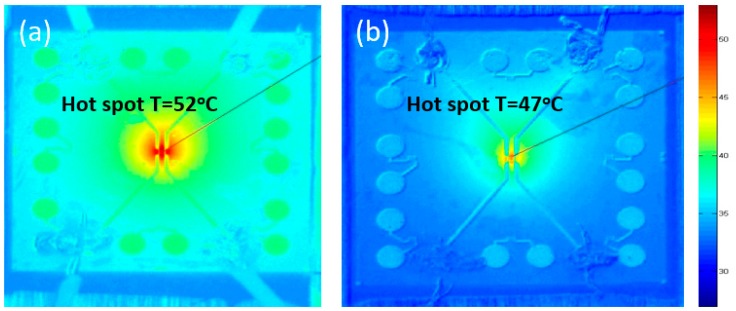
Temperature distributions on thermal test chips at a heat flux of 1280 W/cm^2^ without (**a**) and with (**b**) a graphene heat spreader. Sourced from Reference [38].

In another investigation, an infrared camera was used to monitor the temperature on the thermal test chip to evaluate the cooling performance of a graphene-based heat spreader [38]. The thermal images in Figure 12 show the temperature distributions across the surface of the thermal test chip, which indicate that the temperature decreased by 5 °C when monolayer graphene was used as lateral heat spreader. 

A recent study showed that the cooling performance of a graphene-based heat spreader (fabricated via the vacuum filtration method) can be further improved by interfacial functionalization [36]. In a series of experiments, the graphene films were functionalized by (3-amino-propyl)-triethoxysilane (APTES) molecules to decrease the thermal contact resistance between the graphene-based heat spreader and the hotspot test structure. In this series of experiments, the redesigned resistance thermometer from Figure 9c was used.

**Figure 13:**
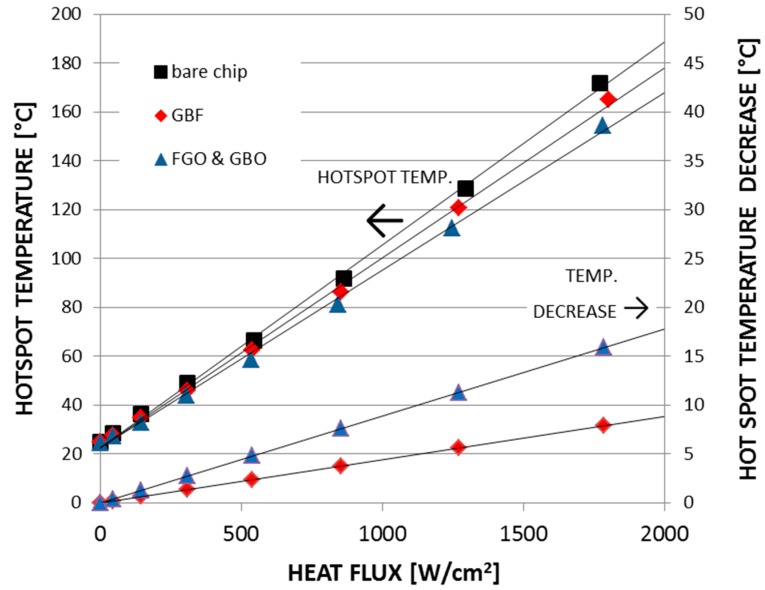
Cooling performance of functionalized graphene-based heat spreaders. Replotted data sourced from Reference [36].

The resulting thermal performance of the graphene-based heat spreader before and after functionalization is shown in Figure 13. It can be seen that the hotspot temperature on a bare chip without graphene heat spreader was 146 °C under a heat flux of 1500 W/cm^2^. By placing a graphene film without functionalization on the surface of the test structure and repeating the measurements, the hotspot temperature was found to decrease to 140 °C (∆T = 6 °C). The estimated accuracy was ±0.5 °C. If, instead, the functionalized graphene-based heat spreader was used, where the thermal contact resistance between the graphene-based film and the test structure was reduced by the addition of a functionalized graphene oxide (FGO) interfacial layer, the hotspot temperature was found to decrease to 134 °C (∆T = 12 °C). 

## 5. Hexagonal Boron Nitride Heat Spreaders

In this paper, we also summarize the use of hotspot test structures for the evaluation of the performance of 2D hexagonal boron nitride (hBN) films as heat spreaders. The advantage of BN films over graphene is that they are electrically insulating and yet good thermal conductors [39]. In scenarios where electrical conduction is not allowed, hBN will be a very good complementary material to graphene for heat spreaders.

Bulk hBN has a typical thermal conductivity of 390 W/mK, which is 280 times higher than the thermal conductivity of silicon dioxide (SiO_2_) insulators. For hBN monolayers, the thermal conductivity value can be even higher [40,41,42]. Thus, the advantage of hBN films is that they might be integrated to the semiconductor circuitry and be placed directly on top or below the hotspot without any insulating SiO_2_ layers, which will significantly decrease the total thermal resistance along the heat conduction path and, therefore, greatly improve the cooling performance. For thermal management applications, 2D hBN was used to develop both thermal composites [43,44,45] and heat spreaders [46,47,48].

In the experiments to be summarized here, hBN films were transferred from the original growth substrate to the hotspot test structure via a similar method as the graphene films [32]. This transfer process includes spin-coating the hBN film with a supporting layer of polymethyl methacrylate (PMMA). The original growth substrate (Cu) was then etched away in a 30% FeCl_3_ solution, leaving the PMMA-supported hBN film floating in FeCl_3_ solution. The monolayer hBN film could then be transferred onto the calibrated hotspot test structure, before the PMMA was dissolved in hot acetone.

**Figure 14:**
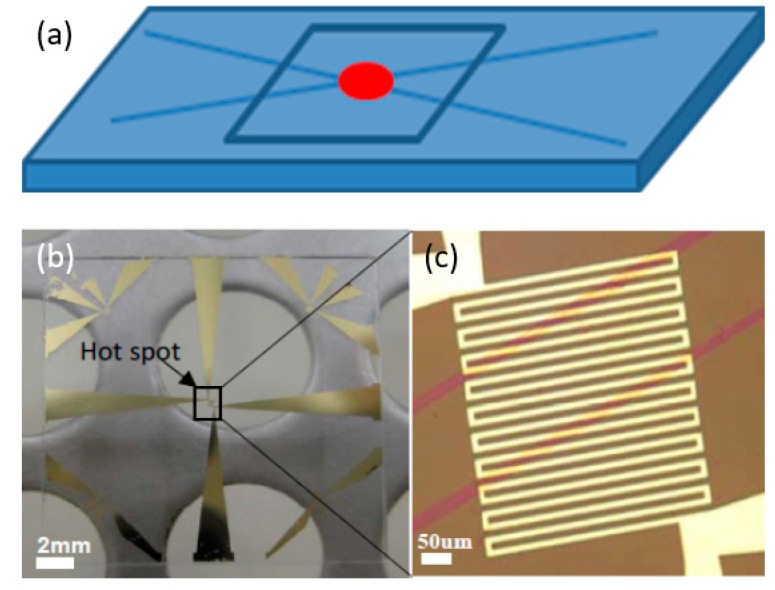
The hotspot test structure with the hexagonal boron nitride (hBN) heat spreader film. Reprinted with permission from [46].

It should be noted that it is very challenging to fabricate freestanding pure hBN films since they are too brittle. Recently, Sun et al. successfully developed a process to fabricate flexible and uniform hBN films by adding acetate cellulose to the hBN dispersion [46]. Before thermal characterization on the hotspot test structure, the quality of the hBN material was examined by TEM. Results showed that few-layer hBN flakes were dominant in the film. The hotspot structure with an hBN heat spreader is shown in Figure 14. Thermal characterization was performed to evaluate the cooling performance of the hBN heat spreader using an infrared camera. Results showed that the hBN heat spreader can lower the hotspot temperature by almost 20 °C under a power density of 625 W/cm^2^.

**Figure 15:**
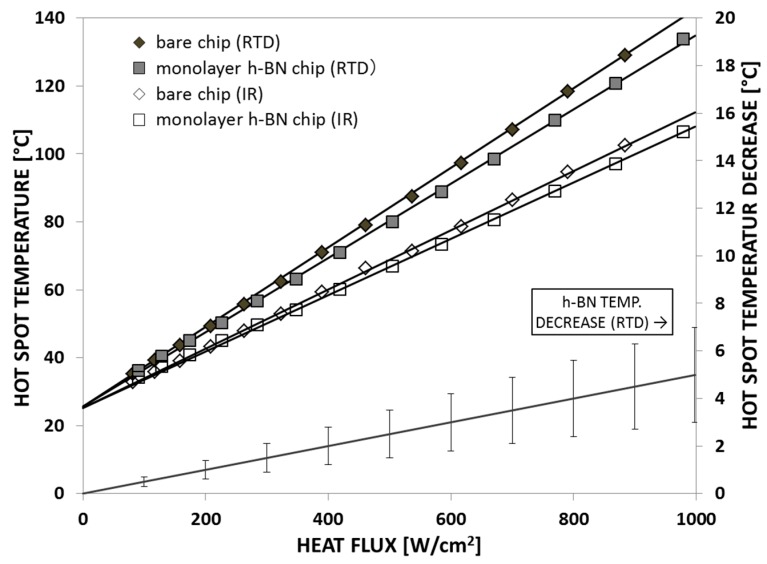
Cooling efficiency of the monolayer hBN heat spreader. Replotted data sourced from Reference [47].

In parallel to this study, Bao et al. applied monolayer hBN films as a lateral heat spreader to cool down the hotspot structure, as shown in Figure 9a [47]. Results showed that the performance of the monolayer hBN heat spreader on the hotspot fabricated on silicon substrates was not as good as in the case of the hotspot fabricated on quartz substrates. This is because a big portion of the heat was conducted through the Si substrate due to its higher thermal conductivity than quartz. Figure 15 shows the hotspot temperature under different power densities. It can be seen that, at a heat flux of 625 W/cm^2^, the hotspot temperature can be reduced by 5 °C. When the heat flux was 1000 W/cm^2^, the hotspot temperature could be reduced by 8 °C using the hBN heat spreader. 

**Figure 16:**
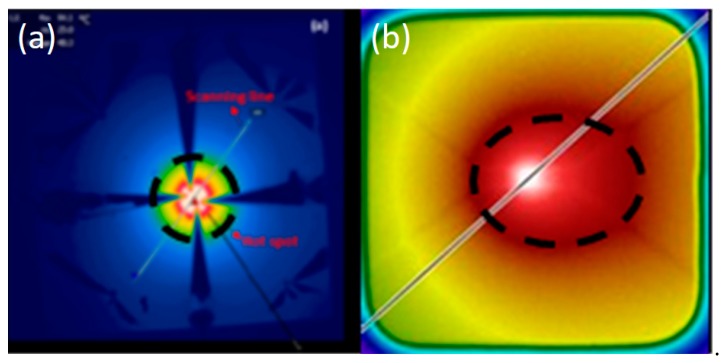
Temperature distribution across the hotspot test chip as captured by infrared camera for two different test structure designs: (**a**) new design; (**b**) old design. Reprinted with permission from Reference [46].

Figure 15 also shows the temperature right below the hotspot (backside of the chip) measured by infrared (IR) camera. An example of such an IR image showing the temperature distribution on the backside of the chip is shown in Figure 16b. This photo again highlights the importance of a proper design of the test structure. The non-negligible resistance of the wires connecting the hotspot resistor with the output pads results in an asymmetrical temperature distribution due to the non-negligible power dissipated in the wires. The temperature distribution can be compared to the one obtained from the improved test structure design used in the previously described experiments. For the IR image captured from the front side of the test chip shown in Figure 16a, which was redesigned with appropriate wire widths and very low power dissipation through the connecting wires, the temperature distribution on the test chip was circular symmetric, which makes it easier to compare with a symmetrical simulation model. 

**Figure 17:**
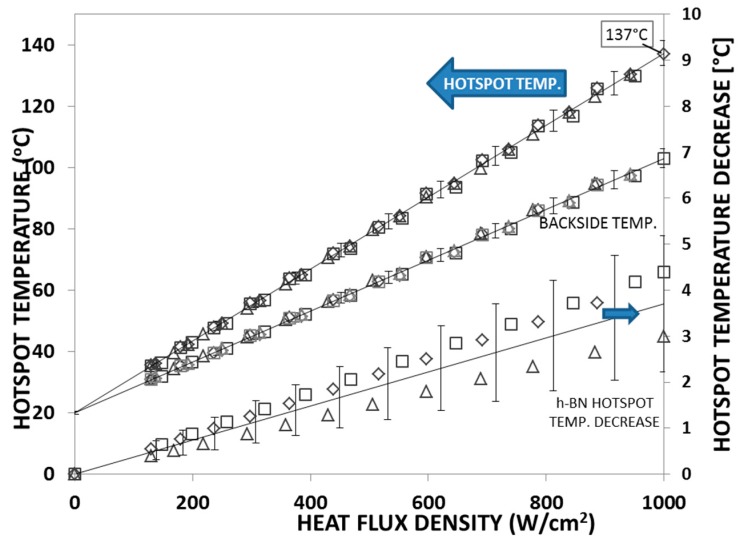
Bare chip hotspot temperature vs. power density by electrical and infrared measurements, as well as the hotspot temperature decrease due to the hBN heat spreader. Replotted data sourced from Reference [48].

For comparison, a similar study was performed using few-layer hBN films obtained from liquid-phase exfoliation (LPE) [48]. In this study, suspension of 2D hBN flakes was prepared with the assistance of sonication in an aqueous surfactant solution containing ethanol. The LPE process lasted 4 h and was followed by 20 min of centrifugation to get rid of the large BN particles. Afterward, the hBN suspension was drop-coated onto the hotspot test structure and then placed on a 60 °C hot plate to evaporate the solvent and obtain the multilayer hBN film as a lateral heat spreader. Details of the fabrication steps can be found in Reference [48]. The results of this study are shown in Figure 17. This graph shows the hotspot temperature vs. power density for three different samples. It can be seen that the temperature decrease at the hotspot was about 3–4 °C at a heat flux of 1000 W/cm^2^—a result somewhat lower than that obtained for monolayer hBN films. 

It should be noted that there is larger variation in thermal performance between different hBN films (multilayer hBN films) as compared to variations between different monolayer hBN films. This is explained by the difficulties in maintaining the same properties between samples obtained by drop-coating of LPE hBN solutions. As shown in Reference [46], studies were also performed where the LPE hBN solution was enhanced by the addition of graphene. 

## 6. Summary and Conclusion

A few emerging low-dimensional materials exhibit excellent thermal properties that could be used for thermal management of high-power electronics. In this paper, we reviewed a number of serpentine hotspot-producing and temperature-sensing test structures that can be used to evaluate the thermal performance of these 1D and 2D materials. The performances of both CNT-based micro heat sinks and two-dimensional films of graphene and hBN-based heat spreaders were summarized. For the CNT-based heat sink, air did not show much cooling effect, while water cooling could lower the hotspot temperature by 50 °C at high heat flux densities. Furthermore, several studies using monolayer graphene and hBN as a heat spreader were summarized. The monolayer graphene heat spreader was shown to be much more efficient in spreading the heat, thereby lowering the hotspot temperature, than the monolayer hBN heat spreader. Concerning few-layer graphene heat spreaders, it was shown that their performance could be improved considerably by functionalization using APTES, which can minimize the thermal contact resistance between the chip and the heat spreader. Few-layer hBN heat spreaders were shown to have similar heat spreading performance to few-layer graphene without functionalization (~5°C at 1000 W/cm^2^).

These 1D and 2D materials show great potential as heat dissipation materials in electronics. However, challenges need to be addressed before the low-dimensional materials can be pushed onto the market. For the CNT-based micro heat sink, a CNT transfer process which can be upscaled to industry level and be compatible with the current semiconductor processes needs to be approved. For graphene-based heat spreaders, the thick graphene films are more favorable than the CVD-grown mono- to few-layer graphene films from the processability perspective. Lastly, hBN-based heat spreaders are easier to integrate into electronic systems than graphene-based heat spreaders because hBN films are electrically insulating; however, the mechanical strength of the hBN films needs to be improved.

## References

[1] Shtein M., Nadiv R., Buzaglo M., Regev O. (2015). Graphene-Based Hybrid Composites for Efficient Thermal Management of Electronic Devices. ACS Appl. Mater. Interfaces..

[2] Xu J., Fisher T.S. (2006). Enhancement of thermal interface materials with carbon nanotube arrays. Int. J. Heat Mass Transfer.

[3] Mo Z., Morjan R., Anderson J., Campbell E.E.B., Liu J. Integrated nanotube microcooler for microelectronics applications. Proceedings of the Electronic Components and Technology.

[4] Kim P., Shi L., Majumdar A., McEuen P.L. (2001). Thermal transport measurements of individual multiwalled nanotubes. Phys. Rev. Lett..

[5] Wang Z., Xie R., Bui C.T., Liu D., Ni X., Li B., Thong J.T.L. (2011). Thermal Transport in Suspended and Supported Few-Layer Graphene. Nano Lett..

[6] Choi D., Poudel N., Park S., Akinwande D., Cronin S.B., Watanabe K., Taniguchi T., Yao Z., Shi L. (2018). Large Reduction of Hot Spot Temperature in Graphene Electronic Devices with Heat-Spreading Hexagonal Boron Nitride. ACS Appl. Mater. Interfaces..

[7] Balandin A.A., Ghosh S., Bao W., Calizo I., Teweldebrhan D., Miao F., Lau C.N. (2008). Superior Thermal Conductivity of Single-Layer Graphene. Nano Lett..

[8] Han H., Zhang Y., Wang N., Samani M.K., Ni Y., Mijbil Z.Y., Edwards M., Xiong S., Sääskilahti K., Murugesan M. (2016). Functionalization mediates heat transport in graphene nanoflakes. Nat. Commun..

[9] Chen Z., Jang W., Bao W., Lau C.N., Dames C. (2009). Thermal contact resistance between graphene and silicon dioxide. Appl. Phys. Lett..

[10] Ramirez S., Chan K., Hernandez R., Recinos E., Hernandez E., Salgado R., Khitun A.G., Garay J.E., Balandin A.A. (2017). Thermal and magnetic properties of nanostructured densified ferrimagnetic composites with graphene - graphite fillers. Mater. Des..

[11] Jeon D., Kim S.H., Choi W., Byon C. (2019). An experimental study on the thermal performance of cellulose-graphene-based thermal interface materials. Int. J. Heat Mass Transf..

[12] Jeppson K., Bao J., Huang S., Zhang Y., Sun S., Fu Y., Liu J. Hotspot test structures for evaluating carbon nanotube microfin coolers and graphene-like heat spreaders. Proceedings of the 2016 International Conference on Microelectronic Test Structures (ICMTS).

[13] Fu Y., Wang T., Jonsson O., Liu J. (2010). Application of through silicon via technology for in situ temperature monitoring on thermal interfaces. J. Micromech. Microeng..

[14] Subrina S., Kotchetkov D., Balandin A.A. (2009). Heat Removal in Silicon-on-Insulator Integrated Circuits With Graphene Lateral Heat Spreaders. IEEE Electron Device Lett..

[15] Nylander A.N., Fu Y., Huang M., Liu J. (2019). Covalent Anchoring of Carbon Nanotube-Based Thermal Interface Materials Using Epoxy-Silane Monolayers. IEEE Trans. Compon. Packag. Manuf. Technol..

[16] Cross R., Cola B.A., Fisher T., Xu X., Gall K., Graham S. (2010). A metallization and bonding approach for high performance carbon nanotube thermal interface materials. Nanotechnology.

[17] Huang H., Liu C.H., Wu Y., Fan S. (2005). Aligned carbon nanotube composite films for thermal management. Adv. Mater..

[18] Biercuk M.J., Llaguno M.C., Radosavljevic M., Hyun J.K., Johnson A.T., Fischer J.E. (2002). Carbon nanotube composites for thermal management. Appl. Phys. Lett..

[19] Lin W., Moon K.S., Wong C.P. (2009). A combined process of in situ functionalization and microwave treatment to achieve ultrasmall thermal expansion of aligned carbon Nanotube-Polymer nanocomposites: Toward applications as thermal interface materials. Adv. Mater..

[20] Lu J.P. (1997). Elastic properties of carbon nanotubes and nanoropes. Phys. Rev. Lett..

[21] Yu M.F., Lourie O., Dyer M.J., Moloni K., Kelly T.F., Ruoff R.S. (2000). Strength and breaking mechanism of multiwalled carbon nanotubes under tensile load. Science.

[22] Fu Y., Nabiollahi N., Wang T., Wang S., Hu Z., Carlberg B., Zhang Y., Wang X., Liu J. (2012). A complete carbon-nanotube-based on-chip cooling solution with very high heat dissipation capacity. Nanotechnology.

[23] Fu Y., Qin Y., Wang T., Chen S., Liu J. (2010). Ultrafast Transfer of Metal-Enhanced Carbon Nanotubes at Low Temperature for Large-Scale Electronics Assembly. Adv. Mater..

[24] Fu Y., Ye L.L., Liu J. (2012). Thick film patterning by lift-off process using double-coated single photoresists. Mater. Lett..

[25] Balandin A.A. (2011). Thermal properties of graphene and nanostructured carbon materials. Nat. Mater..

[26] Shahil K.M., Balandin A.A. (2012). Graphene–Multilayer Graphene Nanocomposites as Highly Efficient Thermal Interface Materials. Nano Lett..

[27] Goli P., Legedza S., Dhar A., Salgado R., Renteria J., Balandin A.A. (2014). Graphene-enhanced hybrid phase change materials for thermal management of Li-ion batteries. J. Power Sources.

[28] Saadah M., Hernandez E., Balandin A. (2017). Thermal Management of Concentrated Multi-Junction Solar Cells with Graphene-Enhanced Thermal Interface Materials. Appl. Sci..

[29] Shtein M., Nadiv R., Buzaglo M., Kahil K., Regev O. (2015). Thermally Conductive Graphene-Polymer Composites: Size, Percolation, and Synergy Effects. Chem. Mater..

[30] Gu J., Xie C., Li H., Dang J., Geng W., Zhang Q. (2014). Thermal percolation behavior of graphene nanoplatelets/polyphenylene sulfide thermal conductivity composites. Polym. Compos..

[31] Li A., Zhang C., Zhang Y.F. (2017). Thermal Conductivity of Graphene-Polymer Composites: Mechanisms, Properties, and Applications. Polymers.

[32] Yan Z., Liu G., Khan J.M., Balandin A.A. (2012). Graphene quilts for thermal management of high-power GaN transistors. Nat. Commun..

[33] Han N., Cuong T.V., Han M., Ryu B.D., Chandramohan S., Park J.B., Kang J.H., Park Y.J., Ko K.B., Kim H.Y. (2013). Improved heat dissipation in gallium nitride light-emitting diodes with embedded graphene oxide pattern. Nat. Commun..

[34] Gao Z., Zhang Y., Fu Y., Yuen M.M., Liu J. (2013). Thermal chemical vapor deposition grown graphene heat spreader for thermal management of hot spots. Carbon.

[35] Gao Z., Zhang Y., Fu Y., Yuen M., Liu J. Graphene heat spreader for thermal management of hot spots in electronic packaging. Proceedings of the 18th International Workshop on THERMal INvestigation of ICs and Systems.

[36] Zhang Y., Han H., Wang N., Zhang P., Fu Y., Murugesan M., Edwards M., Jeppson K., Volz S., Liu J. (2015). Improved Heat Spreading Performance of Functionalized Graphene in Microelectronic Device Application. Adv. Funct. Mater..

[37] Zhang Y., Edwards M., Samani M.K., Logothetis N., Ye L., Fu Y., Jeppson K., Liu J. (2016). Characterization and simulation of liquid phase exfoliated graphene-based films for heat spreading applications. Carbon.

[38] Huang S., Zhang Y., Sun S., Fan X., Wang L., Fu Y., Zhang Y., Liu J. Characterization for graphene as heat spreader using thermal imaging method. Proceedings of the 2013 14th International Conference on Electronic Packaging Technology.

[39] Zhou H., Zhu J., Liu Z., Yan Z., Fan X., Lin J., Wang G., Yan Q., Yu T., Ajayan P.M. (2014). High thermal conductivity of suspended few-layer hexagonal boron nitride sheets. Nano Res..

[40] Lindsay L., Broido D.A. (2012). Theory of thermal transport in multilayer hexagonal boron nitride and nanotubes. Phys. Rev. B.

[41] Ouyang T., Chen Y., Xie Y., Yang K., Bao Z., Zhong J. (2010). Thermal transport in hexagonal boron nitride nanoribbons. Nanotechnology.

[42] Bao J., Jeppson K., Edwards M., Fu Y., Ye L., Lu X., Liu J. (2016). Synthesis and applications of two-dimensional hexagonal boron nitride in electronics manufacturing. Electron. Mater. Lett..

[43] Wang Z., Iizuka T., Kozako M., Ohki Y., Tanaka T. (2011). Development of epoxy/BN composites with high thermal conductivity and sufficient dielectric breakdown strength part I - sample preparations and thermal conductivity. IEEE Trans. Dielectr. Electr. Insul..

[44] Lei Y., Han Z., Ren D., Pan H., Xu M., Liu X. (2018). Design of h-BN-Filled Cyanate/Epoxy Thermal Conductive Composite with Stable Dielectric Properties. Macromol. Res..

[45] Yang N., Zeng X., Lu J., Sun R., Wong C.P. (2018). Effect of chemical functionalization on the thermal conductivity of 2D hexagonal boron nitride. Appl. Phys. Lett..

[46] Sun S., Bao J., Mu W., Fu Y., Zhang Y., Ye L., Liu J. Cooling hot spots by hexagonal boron nitride heat spreaders. Proceedings of the 2015 IEEE 65th Electronic Components and Technology Conference (ECTC).

[47] Bao J., Zhang Y., Huang S., Sun S., Lu X., Fu Y., Liu J. (2016). Application of two-dimensional layered hexagonal boron nitride in chip cooling. J. Basic Sci. Eng..

[48] Bao J., Edwards M., Huang S., Zhang Y., Fu Y., Lu X., Yuan Z., Jeppson K., Liu J. (2016). Two-dimensional hexagonal boron nitride as lateral heat spreader in electrically insulating packaging. J. Phys. D: Appl. Phys..

